# Contrasting chromatin organization of CpG islands and exons in the human genome

**DOI:** 10.1186/gb-2010-11-7-r70

**Published:** 2010-07-05

**Authors:** Jung Kyoon Choi

**Affiliations:** 1 Department of Biology and Brain Engineering, KAIST, 335 Gwahak-ro, Daejeon 305-701, Republic of Korea; 2 Computational and Mathematical Biology, Genome Institute of Singapore, 60 Biopolis Street, Singapore 138672, Republic of Singapore

## Abstract

**Background:**

CpG islands and nucleosome-free regions are both found in promoters. However, their association has never been studied. On the other hand, DNA methylation is absent in promoters but is enriched in gene bodies. Intragenic nucleosomes and their modifications have been recently associated with RNA splicing. Because the function of intragenic DNA methylation remains unclear, I explored the possibility of its involvement in splicing regulation.

**Results:**

Here I show that CpG islands were associated not only with methylation-free promoters but also with nucleosome-free promoters. Nucleosome-free regions were observed only in promoters containing a CpG island. However, the DNA sequences of CpG islands predicted the opposite pattern, implying a limitation of sequence programs for the determination of nucleosome occupancy. In contrast to the methylation-and nucleosome-free states of CpG-island promoters, exons were densely methylated at CpGs and packaged into nucleosomes. Exon-enrichment of DNA methylation was specifically found in spliced exons and in exons with weak splice sites. The enrichment patterns were less pronounced in initial exons and in non-coding exons, potentially reflecting a lower need for their splicing. I also found that nucleosomes, DNA methylation, and H3K36me3 marked the exons of transcripts with low, medium, and high gene expression levels, respectively.

**Conclusions:**

Human promoters containing a CpG island tend to remain nucleosome-free as well as methylation-free. In contrast, exons demonstrate a high degree of methylation and nucleosome occupancy. Exonic DNA methylation seems to function together with exonic nucleosomes and H3K36me3 for the proper splicing of transcripts with different expression levels.

## Background

A CpG island (CGI) is a stretch of DNA in which the frequency of CpGs is higher than that present in other regions [1]. This unique genomic element is found only in vertebrate genomes and is usually present in the promoters of housekeeping genes. CGIs remain typically unmethylated even with many potential target sites for DNA methylation and their aberrant methylation often leads to gene silencing, for example in cancer cells [2].

Gene silencing by DNA methylation is accompanied by local changes in the chromatin structure. A more direct mechanism to regulate chromatin structure is the assembly and disassembly of histone-DNA complexes, or nucleosomes. A hallmark of recent whole-genome profiles of nucleosome positions is the presence of a nucleosome-free region (NFR) in the promoter [3-5]. However, the relationships between the promoter CGI and the NFR remain largely unexplored.

A provocative finding obtained in recent methylome studies is that intragenic DNA methylation occurs at a higher density compared to promoter methylation [6-8], which is suggested to inhibit transcription elongation [9]. Intragenic methylation is associated with neither gene silencing nor a high level of gene expression [6], thereby leaving its biological role an open question.

Recent evidence provides a clue for connections among chromatin structure, RNA polymerase II (pol II) elongation, and RNA splicing. H3K36me3 (trimethylation of Lys36 on histone 3), one of the histone modifications that mark gene bodies, has been shown to be present specifically on constitutively spliced exons of active genes, implicating its role in RNA splicing [10]. The SWI/SNF complex has been suggested to affect RNA splicing by slowing down pol II progression via its chromatin remodeling activity [11]. Likewise, two recent studies have suggested that the exon-specific positioning of intragenic nucleosomes, which function as roadblocks to inhibit pol II, facilitates exon inclusion during RNA splicing [12,13].

Given the suggested links between chromatin regulation and RNA splicing, one might suspect that intragenic DNA methylation plays a similar role, judging by its influence on pol II elongation [9]. Thus, in the present study, I investigated whether CpG methylation was specifically enriched on exons compared to introns and whether it was associated with spliced exons rather than skipped exons, as H3K36me3 and nucleosomes were shown to be.

## Results and discussion

Previous studies have shown that underlying DNA sequences are important determinants of nucleosome occupancy [14,15]. For example, the *in vitro *binding of nucleosomes to naked genomic DNA from different species is dictated in large part by the DNA sequence composition [15]. By collecting nucleosome-bound DNA sequences and center-aligning them, common underlying features of nucleosome-favoring sequences could be found and modeled based on thermodynamics for future predictions of nucleosome formation [14]. In another approach, a support vector machine was employed to build nucleosome prediction models based on different human cell lines [16].

Although promoter sequences have been extensively explored with respect to nucleosome patterns, the mechanism by which CGI sequences affect nucleosome assembly has never been studied. One may postulate that the unique sequence features of CGIs (for example, aberrant high CpG density) may prevent nucleosome assembly, considering the active chromatin structure of CGIs *in vivo *[17].

**Figure 1:**
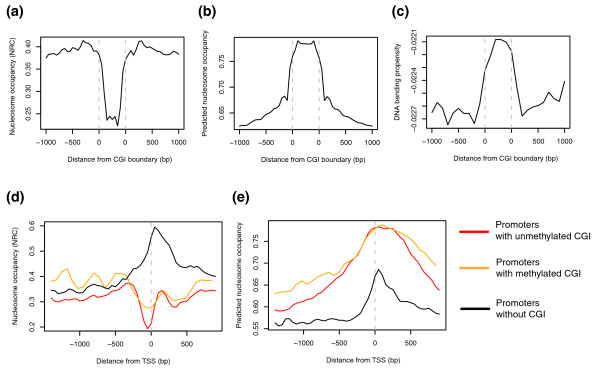
**Nucleosome organization of promoter CGIs**. **(a-c) **Nucleosome patterns upstream, inside and downstream of the CGI (from left to right) based on (a) *in vivo *nucleosome occupancy for human T cells [5] measured as normalized read count (NRC; see Materials and methods), (b) sequence prediction of nucleosome occupancy [15], and (c) DNA bending propensity. **(d,e) **Nucleosome patterns surrounding the transcription start site (TSS) based on (d) *in vivo *nucleosome occupancy for human T cells [5] measured as the NRC and (e) sequence prediction of nucleosome occupancy [15].

Expectedly, the *in vivo *nucleosome occupancy within the CGI is remarkably low compared to that in the flanking regions (Figure 1a). Open chromatin can be identified by DNase I hypersensitivity experiments. I used the whole-genome data of DNase I hypersensitivity sites [18] to assess their enrichment in CGIs (see Materials and methods). The fraction of the human genome that harbors these sites was compared with that of the CGIs that overlap these sites, producing an odds ratio of 14. This means that open chromatin is 14-fold more likely to be found in CGIs than in the other genomic regions.

To assess whether the nucleosome depletion of CGIs is derived from sequence preferences, I utilized the two independent nucleosome prediction datasets mentioned above [15,16]. The portions of the prediction data for CGIs were collected to show that strong nucleosome-favoring features were encoded in the DNA sequences of CGIs (Figure 1b; Additional file 1). This finding is confirmed by the high DNA bendability of CGI sequences, which is required for sharp DNA bending around histone complexes [19] (Figure 1c). The measurement of DNA bending was based on structural parameters that characterize the bending propensity of trinucleotides, as deduced from DNase I digestion data [20].

One factor that can explain this pattern is homopolymeric dA:dT tracts. As important elements in eukaryotic promoters, these tracts are known to act as an intrinsic nucleosome destabilizer [21,22]. Thus, they can be used as a strong indicator of a nucleosome-free state in sequence-based nucleosome prediction models [23,24]. The sequences of CGIs typically lack these elements. A high CG density cannot be maintained in AT-rich sequences. This phenomenon might explain, in part, the nucleosome-favoring signals encoded in CGI sequences.

Reflecting this reciprocal tendency of *in vivo *and predicted nucleosome occupancy, promoters with a CGI tended to maintain a NFR *in vivo *(Figure 1d) against high sequence tendencies toward nucleosome deposition (Figure 1e). Conversely, CGI-lacking promoters exhibited high nucleosome occupancy at the +1 nucleosome location (Figure 1d), which seemed to be programmed by nucleosome sequence preferences (Figure 1e).

The conflicting results obtained from the sequence features and *in vivo *measurements were also demonstrated in the context of DNA methylation. CGIs are typically unmethylated [25,26], notwithstanding many target CpGs in them. It is likely that *trans*-acting regulators are actively recruited to promoter CGIs to maintain this region in a nucleosome-and methylation-free state, overcoming the sequence preferences for high methylation and nucleosome packaging. Accordingly, CGIs showed increased nucleosome occupancy when methylated (orange curve in Figure 1d).

A model of *cis*-programmed nucleosome positioning has been established for the yeast promoters [15]. In the human genome, however, DNA sequences completely fail to predict the presence of promoter NFRs, which is the most distinguishing property of nucleosome positions *in vivo*. This seems due to the unexpected feature of CGIs, which is a conflict between the actions of *cis*-and *trans*-elements in the context of chromatin organization.

CGIs often extend into downstream transcript regions. This provides an explanation for the observation that the exon at the 5' end of the transcript, flanked with the transcription start site, shows a remarkably higher CpG density than the downstream exons (Additional file 2). Given the distinctive chromatin state of CGIs, this might influence exonic nucleosome occupancy and CpG methylation depending on exon location.

**Figure 2:**
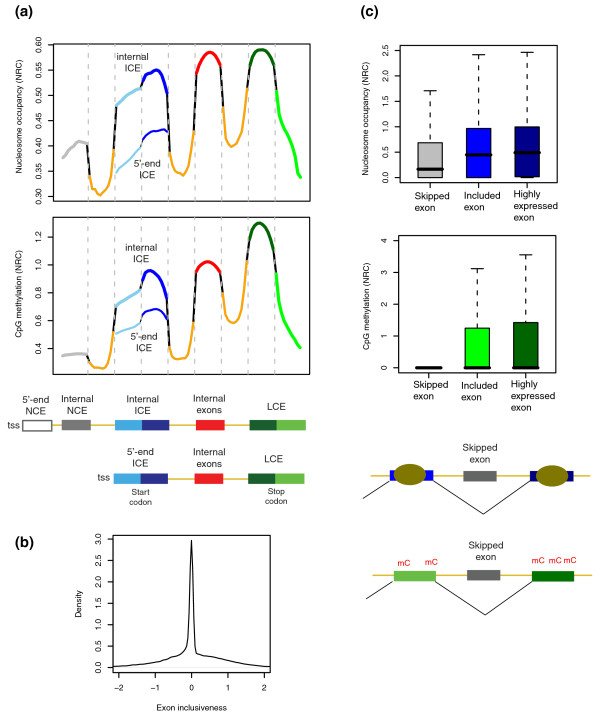
**Exonic DNA methylation and nucleosome occupancy**. **(a) **Nucleosome occupancy (upper panel) and CpG methylation (lower panel) plotted as the average of all transcripts across non-coding exons (NCEs), coding exons, and flanking introns according to their relative positions within the transcript. All exons and introns were partitioned into ten bins and the average normalized read count (NRC) was obtained for each bin of all corresponding exons and introns. ICEs (initial coding exons) and LCEs (last coding exons) are broken into the UTR (light blue or light green) and coding region (dark blue or dark green) by the start codon and stop codon, respectively. The ends of the introns (orange) are connected to those of the flanking exons by the black lines. **(b) **Exon inclusiveness measured as the relative expression of each internal exon compared to the other exons in the transcript. The lowest 10% were considered spliced out and the others to be spliced in. The top 10% were identified as highly expressed for the purpose of checking for sequencing bias. **(c) **Comparison of nucleosome occupancy (upper panel) and CpG methylation (lower panel) among skipped exons, included exons, and highly expressed exons as defined above. tss, transcriptions start site.

An investigation of the DNA methylation and nucleosome occupancy of exons reveals several novel findings (Figure 2a). First, nucleosome occupancy and CpG methylation are enriched in exons relative to introns. Second, non-coding exons (NCEs) show markedly lower enrichment than coding exons, including initial coding exons (ICEs), internal exons, and last coding exons (LCEs). Third, a significant difference is detected between the 5' end ICEs and internal ICEs. Fourth, even though flanking each other within the LCE or ICE, the UTR and the coding region show differential levels of nucleosomes and methylation.

The exonic enrichment of nucleosomes has been reported in most recent studies [12,13]. A similar finding has also been reported for H3K36me3 [10]. Indeed, H3K36me3 showed a pattern similar to that observed for nucleosomes (Additional file 3). The exon enrichment of DNA methylation has been recently reported [27]. A novel observation here is that these marks are differentially distributed among exons with different positions and functions, in a manner that nicely explains their role in RNA splicing.

For example, the 5'-end ICEs do not display high enrichment because they do not require mechanisms for exon inclusion as starting exons only with the splice donor. On the other hand, the functional importance of coding exons might restrict the loss of these marks that ensure exon inclusion into mature transcripts. The maintenance of these marks in coding exons might be assisted by DNA sequence conservation, as indicated by the observation that coding sequences in the ICEs and LCEs show higher enrichment than their flanking UTRs. As compared to 5' UTRs, 3' UTRs are located more remotely from splice acceptors, decreasing the need for these epigenetic mechanisms.

This is the first study to suggest a role for intragenic DNA methylation in RNA splicing. Using the same nucleosome dataset employed herein [5], a previous study has reported the association of high nucleosome occupancy and exons with weak splice sites [13]. Based on the same data for exon strength, I discovered that CpG methylation was also enriched in weak exons (Additional file 4).

Overlapping CGIs on the 5'-end exons seemed to be coupled with a lower level of DNA methylation and nucleosome occupancy (Additional file 2). However, internal NCEs were not affected by CGIs (Additional file 2) but still demonstrated a low level of nucleosome occupancy and CpG methylation similar to introns (Figure 2a). Therefore, it is not likely that the differential enrichment between internal NCEs and internal ICEs results from the CGI effects.

As the methylation data used here were generated based on the affinity of methylation-binding proteins, it is possible that high CpG density on exons results in the exon enrichment of DNA methylation. To resolve this confounding effect, I used the normalized methylation levels divided by CpG density. It seems that CpG density does not affect the DNA methylation patterns (Additional file 5). Another approach to measuring DNA methylation is based on bisulfite treatment, which provides methylation measures on single CpG sites. One such dataset for H1 human embryonic stem cells and IMR90 lung fibroblasts [28] was used and found to reproduce a similar pattern of exon enrichment (Additional file 6).

To further test the role of CpG methylation in RNA splicing, I employed RNA-seq data, which can provide the relative expression of each internal exon compared to the other exons present in the transcript. This measure indicates the inclusiveness of the RNA splicing process for a given exon and is thus termed exon inclusiveness. The exons with the lowest 10% of exon inclusiveness (less than about -1) were considered as spliced out while the others as spliced in. To evaluate sequencing bais, the exons with the top 10% of exon inclusiveness (greater than about 1) were identified as highly expressed (see Materials and methods). The distribution of exon inclusiveness is presented in Figure 2b.

The comparison of nucleosome occupancy and CpG methylation among the above-defined skipped exons, included exons, and highly expressed exons (Figure 2c) reveals that the included exons indeed contain a higher level of epigenetic marks compared to the skipped exons. Moreover, the pattern was not caused by sequencing bias, given the minor differences between the included and highly expressed exons. This result is consistent with the finding that H3K36me3 is enriched on constitutive exons [10] and confirms the hypothesis that these marks can facilitate exon inclusion.

**Figure 3:**
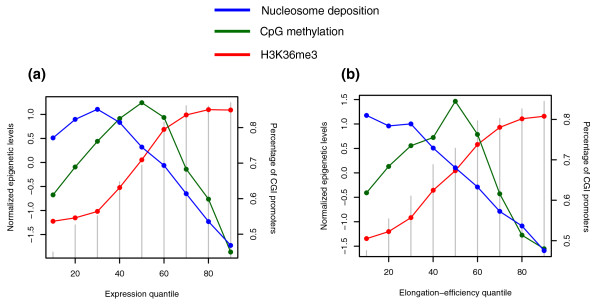
**Normalized nucleosome occupancy, CpG methylation, and H3K36me3 density**. **(a,b) **Normalized nucleosome occupancy, CpG methylation, and H3K36me3 density for internal exons versus **(a) **the quantiles of gene expression level and **(b) **pol II elongation efficiency. The gray lines indicate the percentage of CGI promoters within each bin (y-axis on the right-hand side).

In an effort to find why the three marks are associated with splicing regulation, I discovered that CpG methylation, nucleosome deposition, and H3K36me3 differentially marked the internal exons of genes possessing different expression levels (Figure 3): H3K36me3 marked highly expressed genes as shown in a previous study [10], nucleosomes appeared among lowly expressed genes, and DNA methylation was linked with an intermediate level of gene expression. The elongation efficiency of pol II clarified this pattern (Figure 2b). Genes with a CGI in their promoter tended to be regulated by H3K36me3 rather than nucleosomes or CpG methylation, probably for efficient transcription elongation (see gray lines in Figure 3).

Tilgner *et al. *[13] have shown that when normalized by nucleosome levels, the relative density of H3K36me3 does not show exon-specific enrichment. My hypothesis is as follows. The relative density of H3K36me3 differs between highly and lowly expressed genes. It is the density of nucleosomes that differs between exons and introns. Therefore, the absolute level of H3K36me3, the product of the nucleosome level and the relative modification density, should be different between the exons and introns of highly expressed genes (Additional file 7).

This finding proposes a new model for the influence of epigenetic mechanisms on RNA splicing. Nucleosomes seem to act as roadblocks to pol II passage and expose weak splice acceptors for a long duration to ensure exon inclusion. CpG methylation might play a similar function but with a lower efficiency in pol II inhibition. H3K36me3 appears to accelerate RNA splicing, likely by recruiting the spliceosome-for example, via the CHD1 protein [29]. Although the detailed mechanisms remain to be elucidated, these three marks could function cooperatively to ensure the inclusion of the protein-coding exons of many different transcripts with varying transcriptional activity by differentially controlling pol II elongation efficiency.

In the present study, I focused on the general mechanistic effect of chromatin organization on proper splicing. However, tissue-specific or condition-specific alternative splicing may not be regulated in this way. More elaborate mechanisms involving *cis*-acting RNA sequences and *trans*-acting RNA-binding proteins should accompany this process. Changes in chromatin organization of an exon may result in an alternative inclusion or exclusion of the exon. With epigenomic datasets coupled with RNA profiles for multiple tissues or conditions, we will be able to demonstrate the chromatin regulation of alternative splicing.

## Conclusions

The biological significance of the present findings can be summarized as follows. First, CGIs and NFRs tend to coexist in some promoters, together marking an active chromatin configuration. Only promoters with a CGI tend to display a NFR. In the human genome, promoters lacking a CGI show no evidence of a NFR.

Second, in conflict with *in vivo *nucleosome depletion, the DNA sequences of CGIs encode a strong tendency toward nucleosome formation, highlighting the limitations of DNA sequence programs for the determination of nucleosome positioning.

Third, in support of recent evidence that chromatin regulation mechanisms are linked to RNA splicing, CpG methylation is proposed to cooperate with nucleosomes and H3K36me3 to differentially regulate the elongation of pol II. This finding provides a hint at the role of intragenic DNA methylation, which has remained elusive, and explains why exons maintain the three different mechanisms for their proper splicing.

Fourth, the chromatin regulation of RNA splicing seems to be more intricate than previously considered. The functional importance and DNA sequence constraints of protein-coding exons may explain the dense chromatin organization. The initial exons, which possess splice donors but not acceptors, lack the three marks present in internal and terminal exons.

## Materials and methods

### Measurement of nucleosome occupancy and DNA methylation

H2A.Z-containing nucleosomes in resting human T cells were mapped to the human genome (University of California, Santa Cruz (UCSC) hg18 assembly based on National Center for Biotechnology Information (NCBI) build 36.1) by means of Solexa sequencing technology [5]. The tag coordinate files in the browser extensible data (BED) format for nucleosomes were downloaded from the authors' website [30]. DNA methylation in human T cells was mapped to the human genome by using methyl-CpG-binding domain (MBD) proteins and Solexa sequencing technology [31]. These data are available at NCBI's Gene Expression Omnibus (GEO) repository under accession number [GEO:GSE17554]. The sequencing reads were extended to the average size of fragments in the library (150 bp) [5] and the number of overlapping sequence tags was obtained at 200-bp intervals across the human genome. The ratio of (Target read count/200 bp)/(Total read count/Genome size) was obtained and log2 transformed. This is termed the normalized read count (NRC) and used as an estimate for the DNA methylation level and nucleosomal level at the given genomic locus.

### Measurement of cytosine methylation at base resolution

The degree of methylation at single cytosine nucleotides was measured based on bisulfite treatment for H1 human embryonic stem cells and IMR90 lung fibroblasts [28]. The genomic coordinates of methylated cytosines were downloaded from the authors' website [32]. The ratio between the number of intact cytosines and the total number of intact and bisulfite-converted cytosines was calculated for each locus to indicate the degree of methylation. The cytosines in the CG context were considered.

### Enrichment of open chromatin in CpG islands

A total of 95,723 experimental DNase I hypersensitivity sites for human CD4^+ ^T cells [18] were downloaded from the UCSC genome browser ('dukeDnaseCd4Sites' track). About 80% of the human genome was known to be covered by high-throughput sequencing [33]. The mappable portion of the human genome that harbors open chromatin was compared with the fraction of CGIs that overlap open chromatin, giving rise to an odds ratio indicating the relative enrichment of open chromatin in CGIs.

### Sequence prediction of nucleosome occupancy

Predicted nucleosome level for the human genome (hg18) [15] was downloaded from the authors' website [34]. The average nucleosome occupancy was obtained at 200-bp intervals across the genome. In addition, three different models for human nucleosome prediction [16] were available from the UW Predicted Nucleosome Occupancy track at the UCSC genome browser. The Mec model points to the positions that are frequently nucleosome-free while the A375 and Dennis models indicate those that are frequently occupied by a nucleosome. Again, a model score for each 200-bp genomic interval was obtained. DNA bendability of a given sequence was estimated based on DNase I digestion experiments [20]. Bending parameters for 32 trinucleotides were summed over a target sequence to estimate its DNA bendability.

### Gene expression level and pol II elongation efficiency

Genome-wide gene expression was profiled in resting human T cells by means of DNA microarrays [5], the data for which were available at NCBI's GEO repository under accession number [GEO:GSE10437]. Conceptually, the elongation efficiency of pol II can be calculated as RNA production per unit density of elongating pol II. Transcripts with high elongation efficiency will be produced in high abundance even with a low density of elongating pol II within the transcript. Transcripts with low elongation efficiency will be produced in low abundance even with a high density of elongating pol II within the transcript. Upon transcription initiation, pol II switches to an elongation-competent form with phosphorylation at Ser5 in its carboxy-terminal domain. Thus, elongation efficiency was calculated as the ratio of gene expression level to the density of Ser5-phosphorylated pol II within the transcript body. Genome-wide Ser5-phosphorylated pol II distribution was profiled along with H2A.Z nucleosomes [5] and is available for download from the authors' website [30].

### Detection of skipped exons

RNA-seq was performed by means of Solexa sequencing technology for CD4+ human T cells [35] and the raw sequencing data are available at NCBI's GEO repository under accession number [GEO:GSE16190]. The sequencing reads were extended to the average size of fragments in the library [35] and the number of overlapping sequence tags was obtained at 200-bp intervals across the human genome. The ratio of (Target read count/200 bp)/(Total read count/Genome size) was obtained and log2 transformed. The NRC for each internal exon was obtained and compared with the average read count mapped to all exons of the transcript in question. The difference between the read count of each exon and the average read count of all exons can indicate how inclusive or exclusive the mature transcript is of the given exon. The exons with a large negative difference (lowest 10%), which amounted to > two-fold lower count, were considered to be skipped during splicing in human T cells. The other exons were counted to be included in human T cells. Highly expressed exons - that is, the exons with a large positive difference (highest 10%) - were identified in order to check for sequencing bias. If some genomic regions are easily amplified during Solexa sequencing, high RNA read counts might be inherently correlated with high epigenomic read counts. Without such bias, there will be no significant difference between the set of spliced exons and that of highly expressed exons.

### Calculating the strength of exon splice sites

The sum of the scores of the splice sites of each internal exon was calculated as described in the previous study [13], whereby a total of 76,450 human internal constitutive exons with AG-GT splice sites (50 to 250 bp in length), whose flanking introns were at least 70 bp long and not of U12 type, was used. The lowest scoring 5% and 10% of exons were considered as very weak and weak exons, respectively. Exons with a score greater than the lowest 10% were considered as not-weak exons for control. The average CpG methylation level was calculated for each exon and its flanking intron regions (< 200 bp upstream and downstream of the exon) for the absolute and relative exonic enrichment of CpG methylation.

### CpG islands, exons, and CpG density

The genomic coordinates of CGIs and exons were downloaded from the UCSC genome browser. CpG density was calculated as the ratio of observed to expected CpG frequencies according to the formula cited in Gardiner-Garden and Frommer [36]. CGIs were predicted by the following criteria: GC content of 50% or greater, length greater than 200 bp, and a ratio greater than 0.6 of observed number of CpG dinucleotides to the expected number. A gene was deemed CGI-containing when the region -1,000 bp to 500 bp from the transcription start site overlapped a CGI.

## Abbreviations

bp: base pair; CGI: CpG island; GEP: Gene Expression Omnibus; ICE: initial coding exon; LCCE: last coding exon; NCBI: National Center for Biotechnology Information; NCE: non-coding exon; NFR: nucleosome-free region; NRC: normalized read count; pol II: RNA polymerase II; UCSC: University of California, Santa Cruz; UTR: untranslated region.

## Competing interests

The authors declare that they have no competing interests.

## Authors' contributions

JKC conceived of the study, analyzed the data, and wrote the manuscript.

## Supplementary Material

Additional file 1 **A figure showing nucleosome occupancy upstream, inside and downstream of the CGI as predicted by primary sequences**. Click here for file

Additional file 2 **A figure showing the CpG density of exons with different positioning and their downstream introns**. Click here for file

Additional file 3 **A figure showing the H3K36me3 level observed within the transcript partitioned into non-coding exons, coding exons, and introns**. Click here for file

Additional file 4 **A figure showing specific enrichment of CpG methyaltion on exons with weak splice sites**. Click here for file

Additional file 5 **A figure showing DNA methylation normalized for CpG density within the transcript partitioned into non-coding exons, coding exons, and introns**. Click here for file

Additional file 6 **A figure showing DNA methylation measured at base resolution within the transcript partitioned into non-coding exons, coding exons, and introns**. Click here for file

Additional file 7 **A figure showing a model that explains the higher relative density of H3K36me3 in highly expressed compared to lowly expressed genes, and the higher absolute-level of H3K36me3 in exons compared to introns**. Click here for file

## References

[B1] Bird AP CpG-rich islands and the function of DNA methylation. Nature 1986 321 209 213 10.1038/321209a0 2423876

[B2] Jones PA Baylin SB The fundamental role of epigenetic events in cancer. Nat Rev Genet 2002 3 415 428 10.1038/nrg962 12042769

[B3] Yuan G-C Liu Y-J Dion MF Slack MD Wu LF Altschuler SJ Rando OJ Genome-scale identification of nucleosome positions in *S. cerevisiae*. Science 2005 309 626 630 10.1126/science.1112178 15961632

[B4] Mavrich TN Jiang C Ioshikhes IP Li X Venters BJ Zanton SJ Tomsho LP Qi J Glaser RL Schuster SC Gilmour DS Albert I Pugh BF Nucleosome organization in the *Drosophila *genome. Nature 2008 453 358 362 10.1038/nature06929 18408708 PMC2735122

[B5] Schones DE Cui K Cuddapah S Roh T-Y Barski A Wang Z Wei G Zhao K Dynamic regulation of nucleosome positioning in the human genome. Cell 2008 132 887 898 10.1016/j.cell.2008.02.022 18329373 PMC10894452

[B6] Zilberman D Gehring M Tran RK Ballinger T Henikoff S Genome-wide analysis of *Arabidopsis thaliana *DNA methylation uncovers an interdependence between methylation and transcription. Nat Genet 2006 39 61 69 10.1038/ng1929 17128275

[B7] Zhang X Yazaki J Sundaresan A Cokus S Chan SW-L Chen H Henderson IR Shinn P Pellegrini M Jacobsen SE Genome-wide high-resolution mapping and functional analysis of DNA methylation in *Arabidopsis*. Cell 2006 126 1189 1201 10.1016/j.cell.2006.08.003 16949657

[B8] Cokus SJ Feng S Zhang X Chen Z Merriman B Haudenschild CD Pradhan S Nelson SF Pellegrini M Jacobsen SE Shotgun bisulfite sequencing of the *Arabidopsis *genome reveals DNA methylation patterning. Nature 2008 452 215 219 10.1038/nature06745 18278030 PMC2377394

[B9] Lorincz MC Dickerson DR Schmitt M Groudine M Intragenic DNA methylation alters chromatin structure and elongation efficiency in mammalian cells. Nat Struct Mol Biol 2004 11 1068 1075 10.1038/nsmb840 15467727

[B10] Kolasinska-Zwierz P Down T Latorre I Liu T Liu XS Ahringer J Differential chromatin marking of introns and expressed exons by H3K36me3. Nat Genet 2009 41 376 381 10.1038/ng.322 19182803 PMC2648722

[B11] Batsche E Yaniv M Muchardt C The human SWI/SNF subunit Brm is a regulator of alternative splicing. Nat Struct Mol Biol 2006 13 22 29 10.1038/nsmb1030 16341228

[B12] Schwartz S Meshorer E Ast G Chromatin organization marks exon-intron structure. Nat Struct Mol Biol 2009 16 990 995 10.1038/nsmb.1659 19684600

[B13] Tilgner H Nikolaou C Althammer S Sammeth M Beato M Valcárcel J Guigó R Nucleosome positioning as a determinant of exon recognition. Nat Struct Mol Biol 2009 16 996 1001 10.1038/nsmb.1658 19684599

[B14] Segal E Fondufe-Mittendorf Y Chen L Thåström A Field Y Moore IK Wang J-PZ Widom J A genomic code for nucleosome positioning. Nature 2006 442 772 778 10.1038/nature04979 16862119 PMC2623244

[B15] Kaplan N Moore IK Fondufe-Mittendorf Y Gossett AJ Tillo D Field Y LeProust EM Hughes TR Lieb JD Widom J Segal E The DNA-encoded nucleosome organization of a eukaryotic genome. Nature 2009 458 362 366 10.1038/nature07667 19092803 PMC2658732

[B16] Gupta S Dennis J Thurman RE Kingston R Stamatoyannopoulos JA Noble WS Predicting human nucleosome occupancy from primary sequence. PLoS Comput Biol 2008 4 e1000134 10.1371/journal.pcbi.1000134 18725940 PMC2515632

[B17] Tazi J Bird A Alternative chromatin structure at CpG islands. Cell 1990 60 909 920 10.1016/0092-8674(90)90339-G 2317863

[B18] Boyle AP Davis S Shulha HP Meltzer P Margulies EH Weng Z Furey TS Crawford GE High-resolution mapping and characterization of open chromatin across the genome. Cell 2008 132 311 322 10.1016/j.cell.2007.12.014 18243105 PMC2669738

[B19] Tirosh I Berman J Barkai N The pattern and evolution of yeast promoter bendability. Trends Genet 2007 23 318 321 10.1016/j.tig.2007.03.015 17418911

[B20] Brukner I Sanchez R Suck D Pongor S Sequence-dependent bending propensity of DNA as revealed by DNase I: parameters for trinucleotides. EMBO J 1995 14 1812 1818 7737131 10.1002/j.1460-2075.1995.tb07169.x PMC398274

[B21] Iyer V Struhl K Poly(dA:dT), a ubiquitous promoter element that stimulates transcription via its intrinsic DNA structure. EMBO J 1995 14 2570 2579 7781610 10.1002/j.1460-2075.1995.tb07255.x PMC398371

[B22] Anderson JD Widom J Poly(dA-dT) promoter elements increase the equilibrium accessibility of nucleosomal DNA target sites. Mol Cell Biol 2001 21 3830 3839 10.1128/MCB.21.11.3830-3839.2001 11340174 PMC87046

[B23] Field Y Kaplan N Fondufe-Mittendorf Y Moore IK Sharon E Lubling Y Widom J Segal E Distinct modes of regulation by chromatin encoded through nucleosome positioning signals. PLoS Comput Biol 2008 4 e1000216 10.1371/journal.pcbi.1000216 18989395 PMC2570626

[B24] Segal E Widom J Poly(dA:dT) tracts: major determinants of nucleosome organization. Curr Opin Struct Biol 2009 19 65 71 10.1016/j.sbi.2009.01.004 19208466 PMC2673466

[B25] Bird A DNA methylation patterns and epigenetic memory. Genes Dev 2002 16 6 21 10.1101/gad.947102 11782440

[B26] Yamada Y Watanabe H Miura F Soejima H Uchiyama M Iwasaka T Mukai T Sakaki Y Ito T A comprehensive analysis of allelic methylation status of CpG islands on human chromosome 21q. Genome Res 2004 14 247 266 10.1101/gr.1351604 14762061 PMC327100

[B27] Hodges E Smith AD Kendall J Xuan Z Ravi K Rooks M Zhang MQ Ye K Bhattacharjee A Brizuela L McCombie WR Wigler M Hannon GJ Hicks JB High definition profiling of mammalian DNA methylation by array capture and single molecule bisulfite sequencing. Genome Res 2009 19 1593 1605 10.1101/gr.095190.109 19581485 PMC2752124

[B28] Lister R Pelizzola M Dowen RH Hawkins RD Hon G Tonti-Filippini J Nery JR Lee L Ye Z Ngo Q-M Edsall L Antosiewicz-Bourget J Stewart R Ruotti V Millar AH Thomson JA Ren B Ecker JR Human DNA methylomes at base resolution show widespread epigenomic differences. Nature 2009 462 315 322 10.1038/nature08514 19829295 PMC2857523

[B29] Sims RJ Millhouse S Chen C-F Lewis BA Erdjument-Bromage H Tempst P Manley JL Reinberg D Recognition of trimethylated histone H3 lysine 4 facilitates the recruitment of transcription postinitiation factors and pre-mRNA splicing. Mol Cell 2007 28 665 676 10.1016/j.molcel.2007.11.010 18042460 PMC2276655

[B30] Nucleosome Occupancy and Pol II Distribution Data http://dir.nhlbi.nih.gov/papers/lmi/epigenomes/hgtcellnucleosomes.aspx

[B31] Choi JK Bae J-B Lyu J Kim T-Y Kim Y-J Nucleosome deposition and DNA methylation at coding region boundaries. Genome Biol 2009 10 R89 10.1186/gb-2009-10-9-r89 19723310 PMC2768978

[B32] DNA Methylome Data http://neomorph.salk.edu/human_methylome/data.html

[B33] Rozowsky J Euskirchen G Auerbach RK Zhang ZD Gibson T Bjornson R Carriero N Snyder M Gerstein MB PeakSeq enables systematic scoring of ChIP-seq experiments relative to controls. Nat Biotechnol 2009 27 66 75 10.1038/nbt.1518 19122651 PMC2924752

[B34] Predicted Nucleosome Occupancy Data http://genie.weizmann.ac.il/software/nucleo_genomes.html

[B35] Chepelev I Wei G Tang Q Zhao K Detection of single nucleotide variations in expressed exons of the human genome using RNA-Seq. Nucleic Acids Res 2009 37 e106 10.1093/nar/gkp507 19528076 PMC2760790

[B36] Gardiner-Garden M Frommer M CpG islands in vertebrate genomes. J Mol Biol 1987 196 261 282 10.1016/0022-2836(87)90689-9 3656447

