# Supplementary material for: Contrasting chromatin organization of CpG islands and exons in the human genome
Source: Genome Biol. 2010 Jul 5;11(7):R70. doi: 10.1186/gb-2010-11-7-r70 (PMC2926781; doi:10.1186/gb-2010-11-7-r70)
Supplement: Additional file 1 — A figure showing nucleosome occupancy upstream, inside and downstream of the CGI as predicted by primary sequences. [file gb-2010-11-7-r70-S1.PDF]

Supplementary Fig. 1

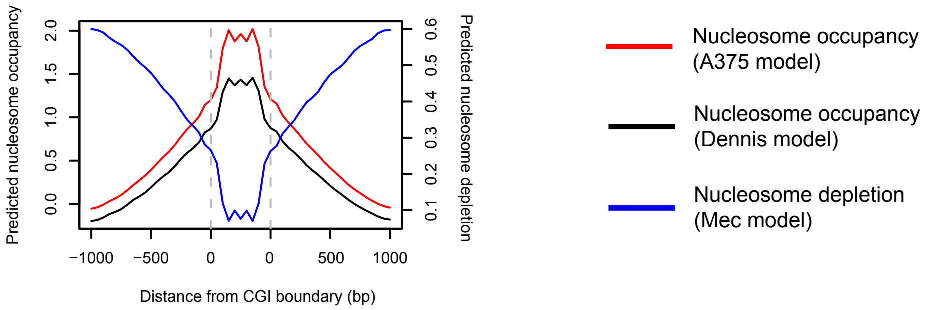

Nucleosome occupancy upstream, inside and downstream of the CGI (from left to right) as predicted by primary sequences. The Mec model points to positions that are frequently nucleosome-free and the A375 model and Dennis model indicate positions that are frequently occupied by a nucleosome (described in ref. 16).
