# Supplementary material for: Contrasting chromatin organization of CpG islands and exons in the human genome
Source: Genome Biol. 2010 Jul 5;11(7):R70. doi: 10.1186/gb-2010-11-7-r70 (PMC2926781; doi:10.1186/gb-2010-11-7-r70)
Supplement: Additional file 2 — A figure showing the CpG density of exons with different positioning and their downstream introns. [file gb-2010-11-7-r70-S2.PDF]

Supplementary Fig. 2

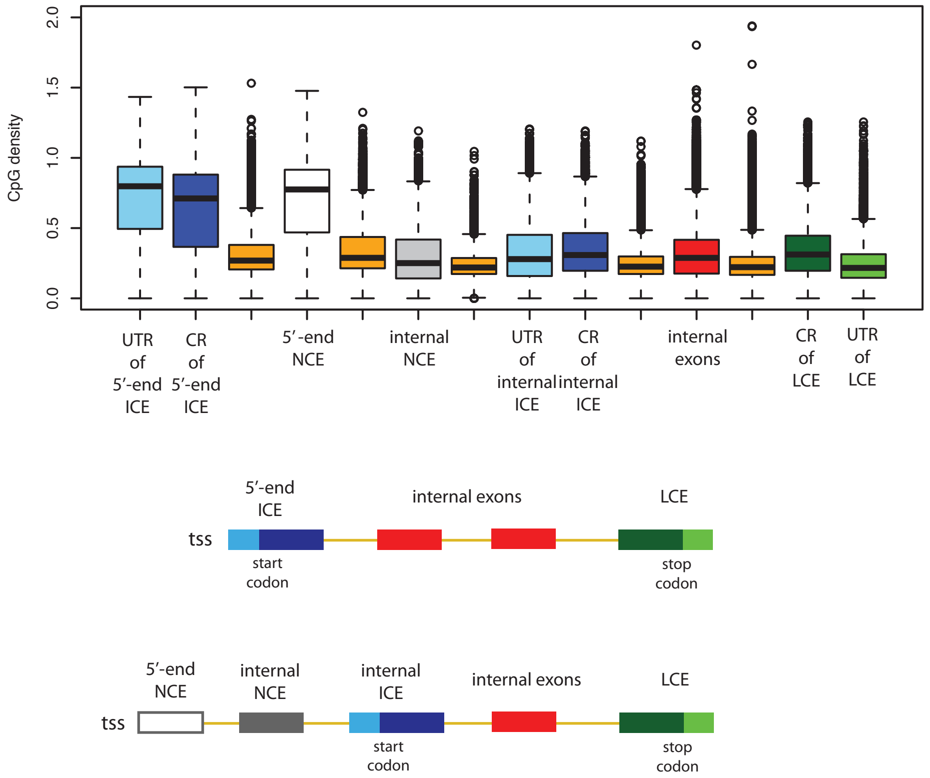

CpG density of exons with different positioning and their follwoing introns.

ICE: initial coding exon, LCE: last coding exon, NCE: non-coding exon, UTR: untranslated region, CR: coding region.
