# Supplementary material for: Contrasting chromatin organization of CpG islands and exons in the human genome
Source: Genome Biol. 2010 Jul 5;11(7):R70. doi: 10.1186/gb-2010-11-7-r70 (PMC2926781; doi:10.1186/gb-2010-11-7-r70)
Supplement: Additional file 3 — A figure showing the H3K36me3 level observed within the transcript partitioned into non-coding exons, coding exons, and introns. [file gb-2010-11-7-r70-S3.PDF]

Supplementary Fig. 3

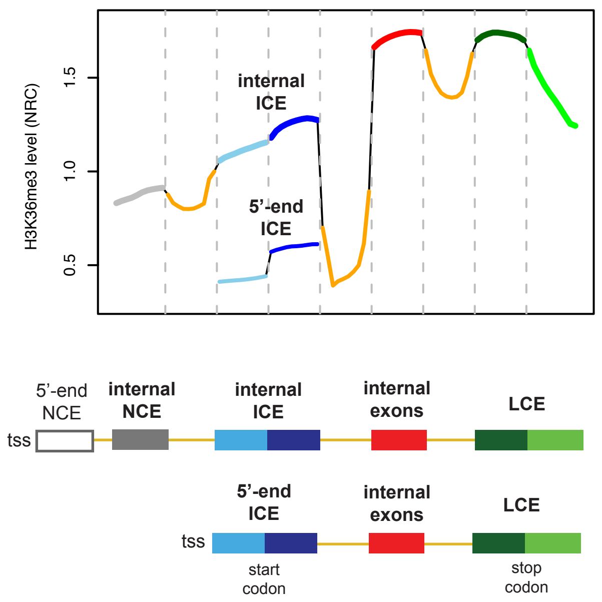

The H3K36me3 level measured as the normalized read count within the transcript partitioned into non-coding exons, coding exons, and introns.

ICEs (initial coding exons) and LESs (last coding exons) are broken into the UTR (light blue or light green) and coding region (dark blue or dark green).
