# Supplementary material for: Contrasting chromatin organization of CpG islands and exons in the human genome
Source: Genome Biol. 2010 Jul 5;11(7):R70. doi: 10.1186/gb-2010-11-7-r70 (PMC2926781; doi:10.1186/gb-2010-11-7-r70)
Supplement: Additional file 4 — A figure showing specific enrichment of CpG methyaltion on exons with weak splice sites. [file gb-2010-11-7-r70-S4.PDF]

Supplementary Fig. 4

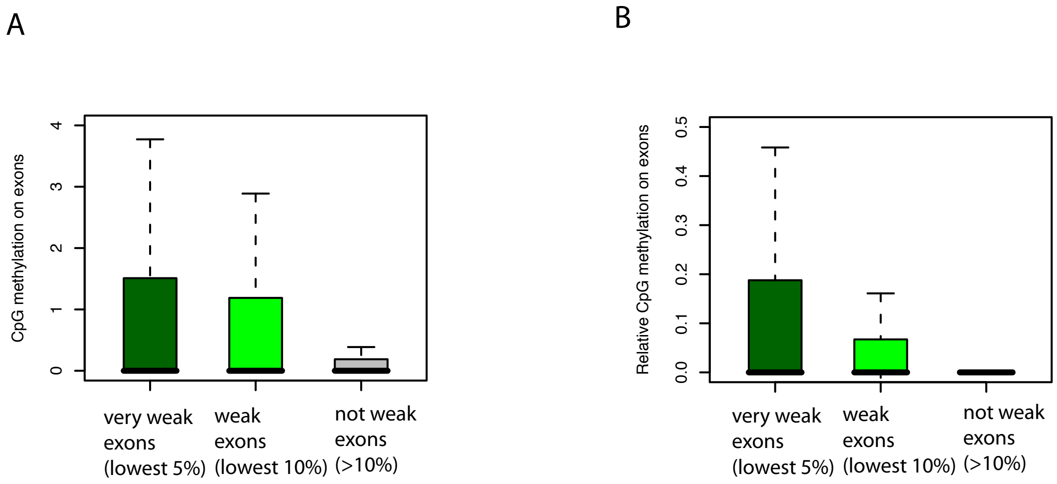

Specific enrichment of CpG methyaltion on exons with weak splice sites

(A) The strength of splice sites was calculated for each exon in the same manner with a previous study (described in Supplementary Methods). CpG methylation level was compared among exons with strength < lowest 5%, those with < lowest 10% and those with > lowest 10% strength.

(B) Relative enrichment of CpG methylation was calculated for each exon in comparison with flanking intron regions (Supplementary Methods). Relative CpG methylation level was compared among exons with lowest 5% strength, those with lowest 10% strength and those with > lowest 10% strength..
