# Supplementary material for: Contrasting chromatin organization of CpG islands and exons in the human genome
Source: Genome Biol. 2010 Jul 5;11(7):R70. doi: 10.1186/gb-2010-11-7-r70 (PMC2926781; doi:10.1186/gb-2010-11-7-r70)
Supplement: Additional file 5 — A figure showing DNA methylation normalized for CpG density within the transcript partitioned into non-coding exons, coding exons, and introns. [file gb-2010-11-7-r70-S5.PDF]

Supplementary Fig. 5

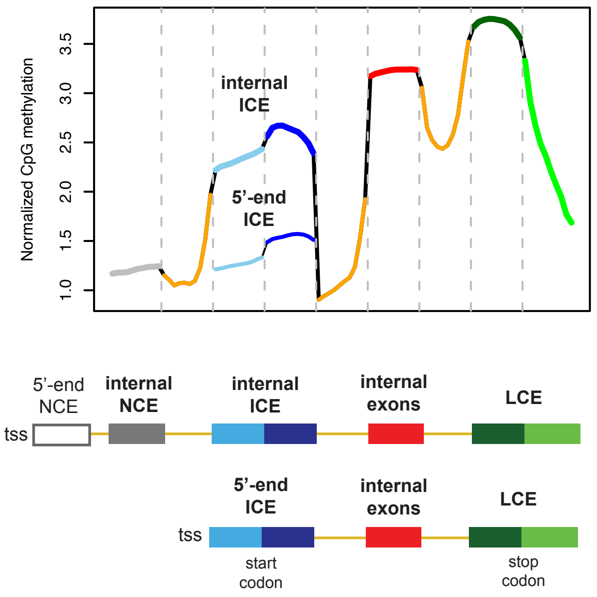

CpG methylation normalized by CpG density on exons and introns.

CpG methylation, measured as the NRC, was divided by CpG density and plotted within the transcript partitioned into non-coding exons, coding exons, and introns.

ICEs (initial coding exons) and LESs (last coding exons) are broken into the UTR (light blue or light green) and coding region (dark blue or dark green).
