# Supplementary material for: Contrasting chromatin organization of CpG islands and exons in the human genome
Source: Genome Biol. 2010 Jul 5;11(7):R70. doi: 10.1186/gb-2010-11-7-r70 (PMC2926781; doi:10.1186/gb-2010-11-7-r70)
Supplement: Additional file 7 — A figure showing a model that explains the higher relative density of H3K36me3 in highly expressed compared to lowly expressed genes, and the higher absolute-level of H3K36me3 in exons compared to introns. [file gb-2010-11-7-r70-S7.PDF]

Supplementary Fig. 7

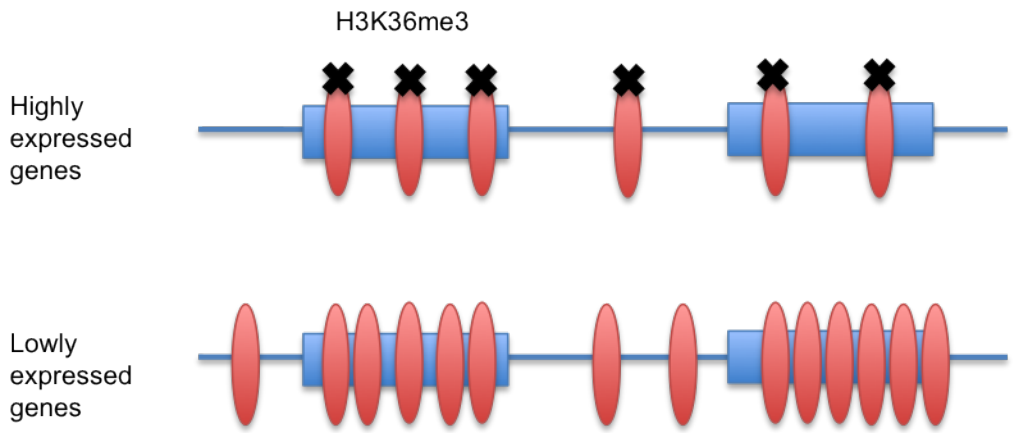

A model that explains the higher relative-density of H3K36me3 in highly-expressed compared to lowly-expressed genes, and the higher absolute-level of H3K36me3 in exons compared to introns.

The relative density means H3K36m3 level per nucleosome while the absolute level means the product of the relative density and the number of nucleosomes
